# Developing technical support and strategic dialogue at the country level to achieve Primary Health Care-based health systems beyond the COVID-19 era

**DOI:** 10.3389/fpubh.2023.1102325

**Published:** 2023-04-11

**Authors:** Jeremy Cheong Chi Mo, Archana Shah, Casey Downey, Sophie Genay-Diliautas, Sohel Saikat, Saqif Mustafa, Nikon Meru, Suraya Dalil, Gerard Schmets, Denis Porignon

**Affiliations:** Special Program for Primary Health Care, Geneva, Switzerland

**Keywords:** Primary Health Care, Universal Health Coverage, health systems strengthening, public health, public health emergency, disaster risk management, global health, World Health Organization

## Abstract

This article is part of the Research Topic ‘Health Systems Recovery in the Context of COVID-19 and Protracted Conflict’.

Pursuing the objectives of the Declaration of Alma-Ata for Primary Health Care (PHC), the World Health Organization (WHO) and global health partners are supporting national authorities to improve governance to build resilient and integrated health systems, including recovery from public health stressors, through the long-term deployment of WHO country senior health policy advisers under the Universal Health Coverage Partnership (UHC Partnership). For over a decade, the UHC Partnership has progressively reinforced, *via* a flexible and bottom-up approach, the WHO’s strategic and technical leadership on Universal Health Coverage, with more than 130 health policy advisers deployed in WHO Country and Regional Offices. This workforce has been described as a crucial asset by WHO Regional and Country Offices in the integration of health systems to enhance their resilience, enabling the WHO offices to strengthen their support of PHC and Universal Health Coverage to Ministries of Health and other national authorities as well as global health partners. Health policy advisers aim to build the technical capacities of national authorities, in order to lead health policy cycles and generate political commitment, evidence, and dialogue for policy-making processes, while creating synergies and harmonization between stakeholders. The policy dialogue at the country level has been instrumental in ensuring a whole-of-society and whole-of-government approach, beyond the health sector, through community engagement and multisectoral actions. Relying on the lessons learned during the 2014–2016 Ebola outbreak in West Africa and in fragile, conflict-affected, and vulnerable settings, health policy advisers played a key role during the COVID-19 pandemic to support countries in health systems response and early recovery. They brought together technical resources to contribute to the COVID-19 response and to ensure the continuity of essential health services, through a PHC approach in health emergencies. This policy and practice review, including from the following country experiences: Colombia, Islamic Republic of Iran, Lao PDR, South Sudan, Timor-Leste, and Ukraine, provides operational and inner perspectives on strategic and technical leadership provided by WHO to assist Member States in strengthening PHC and essential public health functions for resilient health systems. It aims to demonstrate and advise lessons and good practices for other countries in strengthening their health systems.

## Introduction

During the last decades, discussions and debates on how to strengthen health systems in order to operationalize the right to health have been running, without finding a common understanding of how to deliver accessible life-saving health services for all. Despite the commitments expressed in the Declaration of Alma-Ata for Primary Health Care (1) in 1978, reiterated in Astana in 2018 (2), and in the 2030 Agenda for Sustainable Development, as well as significant pieces of evidence linking Primary Health Care (PHC) to improved health outcomes (3), the WHO recently acknowledged that the implementation of PHC has been limited and diverse across countries due to a lack of a universally accepted definition (3).

In 2020, the Operational Framework for Primary Health Care (4) describes it as “a whole-of-society approach to health that aims to maximize the level and distribution of health and well-being through three key components: primary care and essential public health functions as the core of integrated health services; multisectoral policy and actions; and empowered people and communities” (4). It also refers to primary care as a “process in the health system that supports first-contact, accessible, continued, comprehensive and coordinated patient-focused care” (4). During the last few years, WHO’s Member States have committed through several WHO resolutions (5) to use PHC as the fundamental programmatic engine to progress toward the Sustainable Development target 3.8 for Universal Health Coverage (UHC). UHC means that all people have access to the full range of quality health services they need, when and where they need them, without financial hardship.

The comprehensiveness of PHC ensures that any healthcare need is addressed through the direct provision of services at the primary care level or through referral to any other level of care, depending on the package of services defined for each level of the health system. This conceptual framework is broader than the health service delivery function alone and includes essential public health functions (health protection, health promotion, disease prevention, surveillance and response, and emergency preparedness); multisectoral policies to address the social, economic, and environmental determinants of health; and empowering processes to include individuals and communities in the health-related policy-making process.

However, at the beginning of the 2000s, while the largest vertical programs for health were established, disease-specific ventures were more prevalent than integration through health systems strengthening. At this stage, some countries did not develop any national health policy, strategy, or plan for health, and in many others, when elaborated, they were perceived as unrealistic documents and rarely operationalized (6).

From 2000 to 2019, the UHC service coverage has globally increased from 45 to 67 (7) and life expectancy by more than 6 years (8). In the same period until 2017, the maternal mortality ratio dropped by 38% worldwide (9) and the under-5 mortality rate dropped by 60% since 1990 (10). However, 30% of the world’s population are still not able to access the essential health services they need, and almost 2 billion people are facing catastrophic or impoverishing health expenditure (11). Yet, 90% of these needs could be addressed by the PHC approach by providing promotive, preventive, curative, and rehabilitative services accordingly (12, 13). The world has, in consequences, made some great progress on global health; however, further work is still strongly required to reduce inequalities and achieve health for all by 2030.

To build a consensus on how to strengthen health systems, the World Health Organization (WHO) has strongly advocated for the integration of all health programs and functions in the Primary Health Care approach. During the last decades, global public health interventions and emergencies have also demonstrated the need to develop public health policies through an inclusive and multidisciplinary approach to ensure public confidence (14, 15). In addition, many normative documents have been published to develop the PHC approach to health system strengthening.

In 2007, the WHO’s publication on the framework for health systems (16) through the building blocks lens marked a significant change in the admission of the need for an integrated approach, based on the recognition of strong interdependencies between each health system block (17). One year later, while the 2008 World Health Report was making a strong case for PHC (18), the leaders of G8 nations for the first time exchanged on health systems strengthening. In 2009, the World Health Assembly passed a critical resolution that emphasized the importance of Member States’ commitment to “Primary Health Care, including Health System Strengthening” (19). Subsequently, the World Health Report (20) in 2010 outlined how Member States could adapt their health financing system to ensure that all people have access to health services and do not suffer financial hardship paying for them.

In this context, following the 2011 WHA resolutions on strengthening national policy dialogue to build more robust health policies, strategies, and plans (21), the WHO also created the Universal Health Coverage Partnership to enhance governance through policy dialogue with the aim to build resilient and integrated health systems to make progress toward UHC through a Primary Health Care approach. A decade on, the WHO has deployed a large network of more than 130 health policy advisers to support the provision of technical assistance for PHC and UHC in 115 countries. They have been progressively incorporated into the core workforce of WHO to create one of the largest and most effective technical operational platforms and networks for international cooperation on PHC and UHC.

Health policy advisers support policy dialogue and use strategic and technical leadership to enable governments to strengthen health systems, support the harmonization and alignment of partners on National Health Policy and Strategies, and facilitate the implementation of political declarations, such as the one adopted for the High-Level Meeting on UHC during the UN General Assembly in 2019 (22). Furthermore, since 2020, the UHC Partnership has incorporated gender, equity, and human rights components to support the integration of these approaches into national health policies, strategies, and plans based on health inequality and equity monitoring and analysis dimensions.

In 2023, the UHC Partnership channels 10 sources of funds from Belgium, Canada, the European Union, France, Germany, Ireland, Japan, Luxembourg, the United Kingdom, and the WHO. This is to ensure the implementation of its activities and build a bridge between commitments at the global level and national health system strengthening priorities in 115 countries. Funded activities support the WHO’s work plan across all three levels of the organization (country, regional, and headquarters) based on WHO’s Thirteenth General Program of Work 2019–2023 (GPW13), and not as a stand-alone project. The UHC Partnership supports Member States with flexible funds and agile programming while adapting quickly to evolving contexts and priorities.

During the COVID-19 pandemic, Member States benefited from specific assistance to build and maintain sustainable country preparedness and response capacities, including the continuity of essential health services, the integration of innovations, as well as service delivery adaptations in response to COVID-19. Based on country experiences from Colombia, the Islamic Republic of Iran, Lao PDR, South Sudan, Timor-Leste, and Ukraine, in the context of health systems recovery following COVID-19, this policy and practice review provides operational and in-depth perspectives on strategic and technical leadership provided by WHO to assist Member States in strengthening PHC for resilient and integrated health systems.

## Assessment of policy options and implications—Primary health care for resilient and integrated health systems

The COVID-19 pandemic has confirmed that every country is exposed to public health emergencies through direct impact on mortality and morbidity, disruption to health systems functions and essential services, as well as economic and social consequences at the national and global levels. Progress toward UHC and capacities for health security and health determinants are interdependent elements that influence population health. To sustain progress toward UHC, global health security and improved population health and wellbeing require the whole-of-government and social engagement to build the resilience of health systems through health in all policies, considering the complexity of health and the necessity to apply a wide systemic approach (23).

In times of emergencies, health systems are overstretched to respond efficiently to public health threats, while maintaining essential services and functions for the population in dire need. PHC favors integration, coherence, and alignment of health policy and strategies, as well as community engagement, which are critical to ensure that health systems are maintained and continue to deliver services in all contexts. It is also increasingly recognized that facilitating access to PHC is one of the most efficient and convenient ways to increase awareness of menaces to health in the community, by enabling early notification and mitigating and responding to potential threats (24).

Centered on people, PHC brings health systems closer to communities to consider their needs with respect to cultural norms and practices, enhancing trust between health service providers and the population, and also awareness of diseases and care pathways (25, 26). Many essential public health functions, such as surveillance, detection, and notification of diseases, are enhanced through community engagement. Furthermore, compliance with policies cannot be expected as absolute if populations and actors of health systems are not included in policy-making processes, especially in a world fragmented by inequalities (27). Inclusion, solidarity, transparency, and accountability as key components of health system governance are essential for recovering and sustaining progress toward UHC.

The PHC approach to health systems strengthening encompasses these requirements (28–30). The Declaration of Astana is clear about the objectives of PHC: “enhance capacity and infrastructure for primary care (…) prioritizing essential public health functions (…) to meet all people’s health needs across the life course through comprehensive preventive, promotive, curative, rehabilitative services and palliative care” (31). The WHO has translated these resolutions into its 13th General Program of Work (32), recently extended until 2025, and focuses on promoting health, keeping the world safe, and serving the vulnerable.

**Figure 1:**
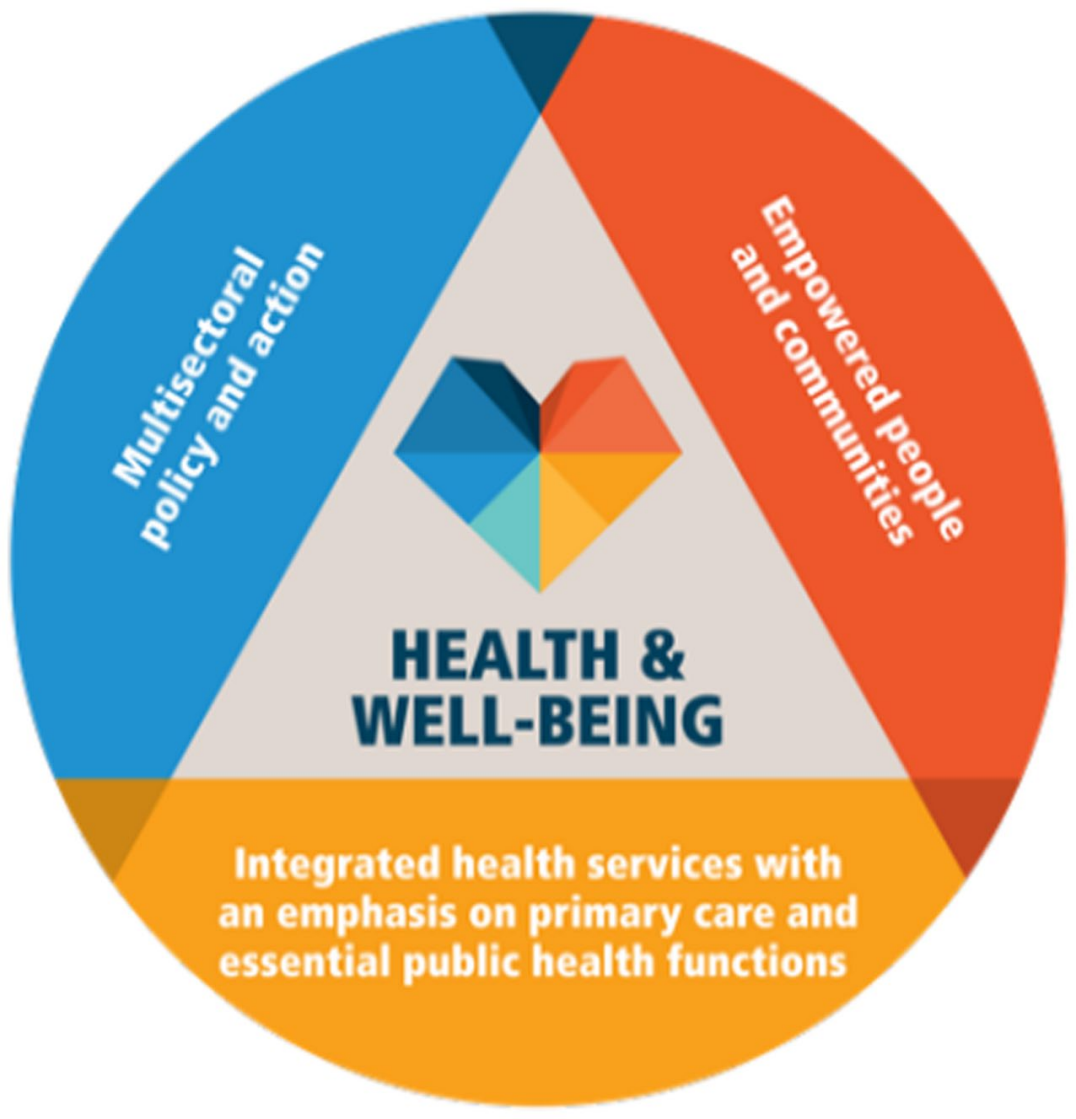
Key components of the PHC approach.

In 2020, the WHO published the Operational Framework for Primary Health Care to clarify the renewed vision of PHC and support countries in scaling up PHC implementation. PHC is defined as a whole-of-government and whole-of-society approach to health that combines, in addition to its focus on primary care and essential public health functions, a strong emphasis on a multisectoral policy and actions perspective, as well as people’s and communities’ empowerment, including private organizations for and not for profit (Figure 1). The operational framework proposes operational and strategic levers to translate PHC commitments into actions. Furthermore, in 2022, a primary healthcare measurement framework and indicators has been published to support Member States to assess, track, and monitor PHC performance to accelerate progress toward UHC and the health-related SDGs (33).

Health systems must be integrated and oriented toward PHC as the foundation for UHC and health security. The COVID-19 pandemic has kept the doors open to implementing PHC as one of the best ways to ensure progress toward UHC and health security (23).

Similarly, prioritization of preparedness and response capacities, or vertical disease programs, without considering building basic health systems functions, cannot deliver the essential health services required by the people. Health system integration can be considered as horizontal to cover a continuum of health services through a single delivery platform, and as vertical to ensure the coordination between platforms of health service delivery, such as between primary and referral care to hospitals, or between public and private, for and not for profit health facilities. Primary care facilities are keeping the gate and maintaining the path to specialty care and hospital care.

## Methodology to analyze the role and the impact of the UHC partnership

Complexity is a significant element of the difficulty to demonstrate and comprehensively understand the results and effects of the intervention of the UHC Partnership (34, 35). Scholars and public health professionals recognized widely that evaluating complex interventions, especially when randomized controlled trials are not feasible, requires to use “non-experimental, mixed methods and process-based approach, appreciation of the different logics of causality, and use of case study research to understand context” (36).

To analyze the role and impact of the UHC Partnership in countries, a formative evaluation was conducted in 2016. (37) It focused on its actions that focus on lessons learned with regard to its role (convener, broker, and technical assistance), strengths (flexibility, bottom-up approach, seed/catalytic funding, and WHO’s Joint Working Team three-level agile network approach), and weaknesses (roster of technical assistance and difficulties finding appropriate candidates).

**Figure 2:**
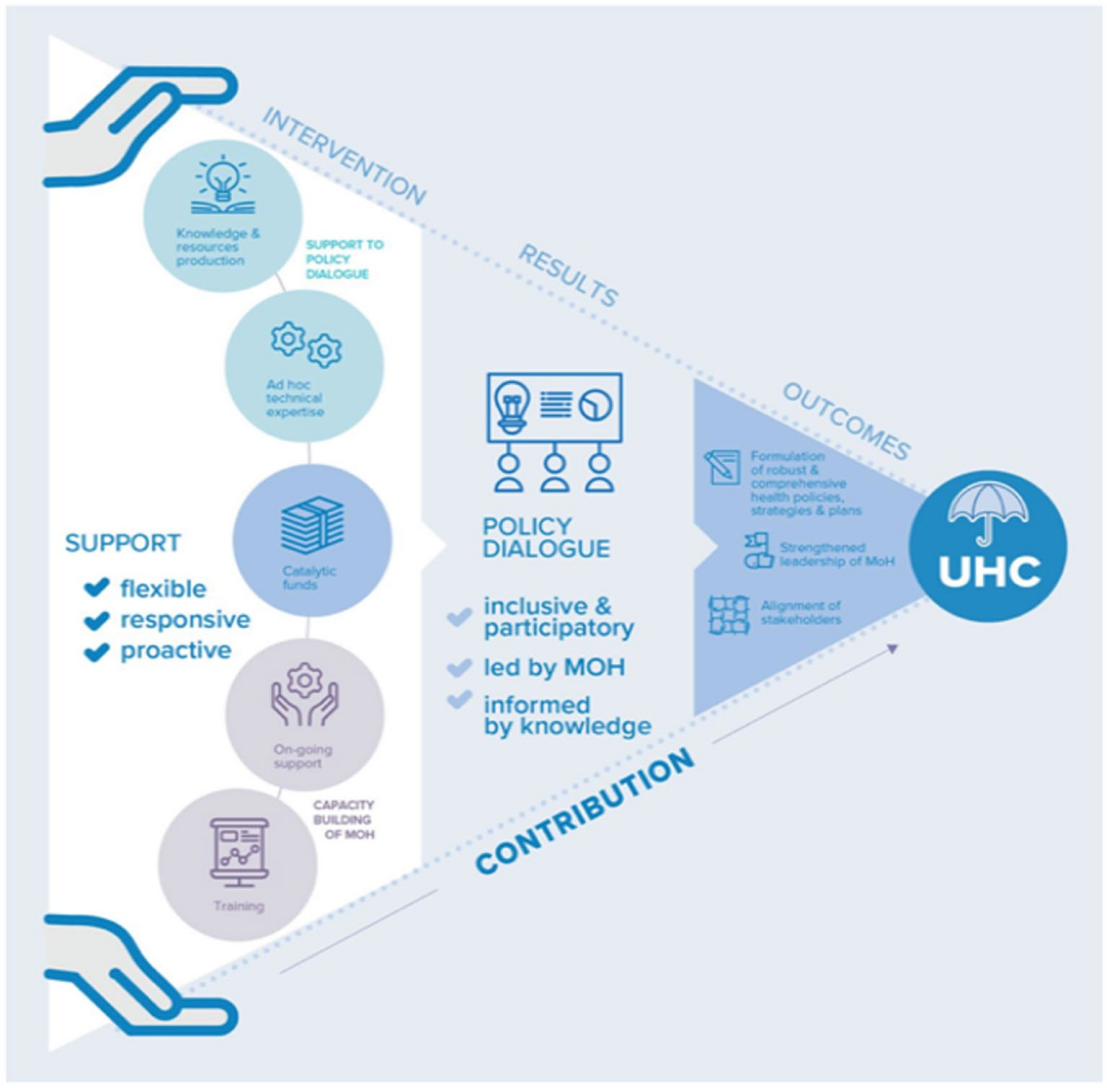
UHC Partnership theory highlighted by the realist evaluation.

In addition, a research approach was also initiated which led to a protocol for a realist evaluation aiming at analyzing policy dialogue processes in their context to understand what mechanisms have triggered health systems to move toward achieving UHC (38). The results report the theory of the underlying rationale of the WHO through the UHC Partnership (Figure 2) which supports the Ministries of Health (MoH) to lead inclusive, participatory, and evidence-informed policy dialogue (39). The support of the health policy advisers should result in mutual trust to strengthen stakeholders’ collaboration, while the evidence and data provided should bring a shared understanding of needs and policy options. The evaluation also reveals the necessary conditions for successful policy dialogue such as dynamic local stakeholders, promotion of collaboration as a mode of action, involvement and leadership of the Ministry of Health, and synergy of messages and actions of WHO. The African Regional Office also published lessons learned on health policy dialogue led within the continent in the frame of the UHC Partnership (40).

To better understand the effects of the interventions, the implementation of activities and results achieved have been described in a systematic manner (41) since the initiation of the UHC Partnership, through annual reports or diverse strategic and technical analytical deep dives. Furthermore, to improve transparency and mutual accountability, and ensure systematic monitoring of implementation and progress, as well as continuity and stability of the efforts at the national level, the UHC Partnership is established through a high-level governance structure and operational pillars.

The governance structure has two key oversight committees: a Multi-Donor Coordination Committee and a WHO high-level UHC Partnership Steering Committee. The operational pillar is composed of the live-monitoring mechanism; the communication and advocacy strategy; as well as the strategic and operational platform named the three-level Joint Working Team for PHC and UHC. All these mechanisms combined provide various opportunities for WHO and partners to actively engage in a regular dialogue on the provision of support to Member States and results achieved to deliver on their UHC goals.

This policy and practice review is a first attempt to formulate what has been observed over time through these diverse accountability mechanisms, in the frame of a larger contribution analysis (42) that should be implemented in the next phase of the UHC Partnership. Country examples have been selected to reflect the diversity of context where the UHC-P is operating, representing each of the six WHO regions, with a long engagement in four low-income countries (Lao PDR, South Sudan, Timor-Leste, and Ukraine) and a shorter one in two middle-income countries (Colombia and the Islamic Republic of Iran). This diversity of context also includes interventions in fragile and conflict-affected countries (South Sudan and Ukraine).

Each case study has been reported in two steps. First, country data for the tracer indicators 3.8.1 and 3.8.2 have been collected to observe the country’s progress toward Universal Health Coverage. These quantitative indicators have been selected because they best reflect the ultimate objective of the UHC Partnership, to increase the coverage of health services and decrease catastrophic health expenditures. Two separate metrics are used to follow this objective, specifically indicator 3.8.1 on the coverage of essential health services and indicator 3.8.2 on catastrophic health spending.

The coverage of essential health services (3.8.1) is defined as the average coverage of essential services based on tracer interventions that include reproductive, maternal, newborn and child health, infectious diseases, non-communicable diseases, and service capacity and access, among the general and the most disadvantaged population. The indicator is measured as an index reported on a unitless scale of 0–100, which is computed as the geometric mean of 14 tracer indicators of health service coverage.

The proportion of the population with household expenditures on health >10% of total household expenditure or income (3.8.2) is estimated as the population-weighted average of the country-level share of people with such catastrophic health expenditures (10% threshold) for a reference year. Incidence at the country level for the reference year is estimated using different methods depending upon the availability of information for that country around or at the reference year.

In a second phase, the gray literature produced by the UHC Partnership (annual reports, evaluations, communication and advocacy documents, policy briefs, blog publications, and online presentations) has been reviewed to list qualitative and quantitative information that could support the establishment of a clear theory of change (activities supported, evidence generated, and output and outcome achieved) to explain the contribution of the UHC Partnership to the achievement of the tracer indicators 3.8.1 and 3.8.2.

The general hypothesis, articulated through this article, supposes that the health system guidance supported by the UHC Partnership aims at elaborating public health policies for UHC with a PHC approach, through policy dialogue while supporting the alignment of financial and human resources and coordinating national and international health partners. It is assumed that it can lead to improve health outcomes and outputs as described in the WHO GPW13 and to improve the tracer indicators 3.8.1 and 3.8.2.

Demonstrating the attribution of results from the technical support provided by the WHO to improve the leadership of both WHO and MoH, as well as governance of health systems and essential public health functions is challenging. This is because governance is complex and involves many different actors, spaces, and parameters, in many different contexts, where it is not straightforward to simply articulate how many lives have been saved because of the development of public health policies or the improvement of strategic frameworks for the health sector in a country.

If quantitative studies demonstrated a positive association between better governance and better health outcome through statistical analysis (43, 44), policy studies are not yet able to provide a reasonable and comprehensive theory that can explain with causality relations the different mechanisms leading to better health outcomes through governance. As the WHO and the Alliance for Health Policy and System Research stated in 2013: “despite abundant evidence of the efficacy of affordable, life-saving interventions, there is little understanding of how to deliver those interventions effectively in diverse settings and within the wide range of existing health systems” (45).

This policy and practice paper seeks to contribute to a plausible understanding of how to strengthen the health system by developing, negotiating, implementing, monitoring, and evaluating robust and integrated national health policies oriented toward UHC. It will also determine if the available evidence is sufficient, and if further investigations would be required, to establish strong theories of change in each country to explain the contribution of the UHC Partnership to achieve Universal Health Coverage.

## Results

Acting on lessons learned during the 2014–2016 Ebola Outbreaks in West Africa and from fragile, conflict-affected, and vulnerable settings (FCV), health policy advisers have been critical during the COVID-19 pandemic to support health systems’ early recovery, to ensure the continuity of essential health services, and to strengthen PHC for health security, including surveillance and treatment of diseases and preventing routine local outbreaks from becoming larger disruptive emergencies. Due to the flexibility of the planification process, they have been able to adapt their support to the new context of the response to the pandemic and its socioeconomic consequences. Several country experiences have been selected among the 115 countries supported in 2022. They are introduced below to describe how increased PHC can support the resilience of health systems (Table 1).

**Table 1:** Country examples of technical assistance for resilient and integrated health systems (46–48).

**Colombia** *Population – 51,265,841 (2021)* *Income level – UMIC* *HDI Index – 0.752 (2021)* *WHO support modalities – Strategic support to institutional transformation* *UHC Partnership Member for 3 years*	UHC Service Coverage Index (SDG 3.8.1)					
	**2000**	**2005**	**2010**	**2015**	**2017**	**2019**
	51	64	69	76	77	78
	Population with household expenditures on health >10% of total household expenditure or income (SDG 3.8.2) (%)					
	**1997**		**2008**		**2016**	
	21,31		20,01		8,19	
	**UHC Partnership actions during the COVID-19 pandemic** While COVID-19 was spreading across Colombia, the country tried to prevent widespread transmissions in areas like the Alta Guajira desert, a remote region inhabited by some of the most vulnerable communities in the country. With the technical support of the health policy adviser, the Government has been enhancing access to primary health care that respects indigenous cultures and traditions to protect them from the pandemic and address common health conditions such as malnutrition, acute diarrheal disease, tuberculosis, acute respiratory diseases and maternal and neonatal morbidities and mortality. An intercultural health model has been implemented based on community health workers. Native and well trained, they are the best positioned to respect cultures, identify health risks and refer to appropriate services. Their close proximity with communities is also a substantial advantage to facilitate the early recovery of the health system. In addition, under COVID-19 guidance, all communities across Colombia were obliged to cremate people when they die, but an exception was made for the indigenous people of Alta Guajira while establishing a clear protocol to ensure the safety of populations.					
**Islamic Republic of Iran** *Population – 85,028,760 (2021)* *Income level – UMIC* *HDI Index – 0.774 (2021)* *WHO support modalities – Strategic support to institutional transformation* *UHC Partnership Member for 2 years*	UHC Service Coverage Index (SDG 3.8.1)					
	**2000**	**2005**	**2010**	**2015**	**2017**	**2019**
	37	49	57	69	74	77
	Population with household expenditures on health >10% of total household expenditure or income (SDG 3.8.2) (%)					
	**2005**	**2010**	**2015**	**2017**	**2018**	**2019**
	11,31	13,72	17,03	16,86	17	15,35
	**UHC Partnership actions before the COVID-19 pandemic** Since 2020, the Islamic Republic of Iran benefits from the presence of a dedicated health policy adviser who supports the operationalization of Primary Health Care. “Each home one health post” is the name of the national PHC initiative implemented by the Ministry of Health to bring health and care closer to communities. A strong network of Primary Care facilities and community health workers serves as the first point of contact for communities. **UHC Partnership actions during the COVID-19 pandemic** Initially aimed to strengthen prevention and health promotion, the program has been crucial in the context of COVID-19 to raise awareness, support early case detection, contact tracing, triage and referral to hospitals. The health policy adviser assisted the Ministry of Health and Medical Education to pilot and scale up a PHC measurement and improvement model and accelerate the national response to COVID-19. Under this platform, assessments and analyses have been produced to implement changes and strengthen PHC. Primary care facilities were also supported to improve health literacy and health promotion by developing training packages, conducting virtual training and by engaging the public. They were critical to reduce overcrowding in hospitals, while continuing to provide essential health services.					
**Lao PDR** *Population – 7,379,358* *Income level – LMIC* *HDI Index – 0.607 (2021)* *WHO support modalities – Technical assistance to strengthen health system foundations* *UHC Partnership Member for 8 years*	UHC Service Coverage Index (SDG 3.8.1)					
	**2000**	**2005**	**2010**	**2015**	**2017**	**2019**
	26	34	39	45	48	50
	Population with household expenditures on health >10% of total household expenditure or income (SDG 3.8.2) (%)					
	**2002**			**2007**		
	3,07			2,98		
	**UHC Partnership actions during the COVID-19 pandemic** In the People’s Democratic Republic of Lao, the COVID-19 pandemic was increasingly affecting the mental health of the population either directly due to illness or due to economic hardships they experienced as a result. Over 95% of people with serious mental illness are untreated, and access to mental health facilities is uneven across the country. Out of the total health workforce, only 42 personnel were working in mental health facilities in the country. Following several emergencies in the past years, the Ministry of Health understood that mental health and psychosocial support is a critical part of any recovery phase, and especially with COVID-19 plan. **UHC Partnership actions beyond the COVID-19 pandemic** Primary care was identified as the best level to improve mental well-being and promotion in villages. The core of the strategy was to enhance the capacities of the existing workforce to deliver mental health services. Through the health policy adviser, the Ministry of Health engaged in the WHO’s Mental Health GAP program to scale up mental health services (development of mental health and psychosocial support guidelines, trainings at all levels). The integration of mental health services with primary care is essential to ensure their availability whenever and wherever people need them.					
**South Sudan** *Population – 11,381,377 (2021)* *Income level – LIC* *HDI Index – 0.385 (2021)* *WHO support modalities – Technical assistance to strengthen health system foundations* *UHC Partnership Member for 10 years*	UHC Service Coverage Index (SDG 3.8.1)					
	**2000**	**2005**	**2010**	**2015**	**2017**	**2019**
	20	21	24	28	31	32
	Population with household expenditures on health >10% of total household expenditure or income (SDG 3.8.2) (%)					
	**2009**		**2016**		**2017**	
	8,72		11,71		13,37	
	**UHC Partnership actions before the COVID-19 pandemic** Since 2018, after 5 years of war, South Sudan is in a transition phase, as its government moved from a core focus of tackling a humanitarian and emergency situation toward reorienting the state’s priorities to long-term development of the health sector. It is one of the first fragile, conflict-affected and vulnerable context country which has been supported by the WHO to develop a health sector stabilization and recovery plan (HSSRP 2020–2022). The health policy adviser played a convening and brokering role by Ministry of Health to coordinate partners and developed an investment plan on catalytic actions to foster the recovery, growth and performance of the health system. This allowed better bridging between humanitarian, emergencies and development partners and increased synergies around the PHC strategic and operational levers. **UHC Partnership actions beyond the COVID-19 pandemic** As part of WHO’s support, through a year-long funded project, the Ministry of Health implemented a PHC project in four states (Jonglei, Western Bahr el Ghazal, Eastern Equatorial and Central Equatorial) with the technical support provided by the health policy adviser. The project was established after the development of the HSSRP and aimed to address critical gaps in health systems foundations, across all essential public health functions, to create a more enabling environment for the advancement of PHC. To achieve this, an integrated approach was applied to synergize efforts related to health systems strengthening, emergency preparedness and response and essential health services delivery. This includes emphasis on health services to vulnerable groups – particularly women, girls, infants and under five children – and strengthening the country’s capacity for early warning, risk reduction and effective management of public health risks.					
**Timor Leste** *Population – 1,343,875 (2021)* *Income level – LMIC* *HDI Index – 0.607 (2021)* *WHO support modalities – Technical assistance for institutional* *transformation UHC Partnership Member for 10 years*	UHC Service Coverage Index (SDG 3.8.1)					
	**2000**	**2005**	**2010**	**2015**	**2017**	**2019**
	33	32	46	49	50	53
	Population with household expenditures on health >10% of total household expenditure or income (SDG 3.8.2) (%)					
	**2001**		**2007**		**2014**	
	2,59		2,36		2,61	
	**UHC Partnership actions before the COVID-19 pandemic** For almost 10 years, the Ministry of Health has benefited from technical assistance to strengthen its governance toward Primary Health Care based health system for UHC, including health financing and human resources for health. The government established legal frameworks to promote inclusive decision-making processes and improve communities’ representation. Thanks to the presence of a health policy adviser, the national health sector governance was strengthened through the establishment of protocols and procedures for partnership and governance (multisectoral policy dialogues and partners coordination mechanism), and the revision of national health strategies (2011–2030 National Health Sector Plan, National Action Plan for Health Security, Human Resources Strategy for PHC). Additionally, WHO provided strong support during the elaboration of a comprehensive service package for PHC through the “Saude na Familia,” the national program for PHC. **UHC Partnership actions beyond the COVID-19 pandemic** When the COVID-19 started to affect the country, the Government scaled up its investments in PHC to strengthen social protection, close gender gaps and related inequalities and enhance digital connectivity. Within 5–6 weeks, it transformed to have in-country testing, functional COVID-19 facilities, staff rapidly trained on COVID-19 management, a gradual increase in stocks of personal protective equipment (PPE), expanded capacity for an expanded testing strategy and active surveillance capabilities. WHO’s previous work with Timor-Leste on governance and emergency preparedness paved the way for an effective response and coordinated and coherent support from health partners to meet the government’s needs including additional funding.					
**Ukraine** *Population – 43,814,581 (2021)* *Income level – LMIC* *HDI Index – 0.773 (2021)* *WHO support modalities – Technical assistance for institutional transformation* *UHC Partnership Member for 8 years*	UHC Service Coverage Index (SDG 3.8.1)					
	**2000**	**2005**	**2010**	**2015**	**2017**	**2019**
	48	51	59	63	70	73
	Population with household expenditures on health >10% of total household expenditure or income (SDG 3.8.2) (%)					
	**2002**	**2005**	**2010**	**2015**	**2017**	**2019**
	12,41	8,2	6,91	7,13	7,3	8,32
	**UHC Partnership actions before the COVID-19 pandemic** Since 2014, Ukraine has been implementing one of the most ambitious programs of reform for Primary Health Care with the technical assistance of a dedicated health policy adviser. The WHO has been a strong supporter especially with regards to the health financing reform in 2016, the new public health legal framework and the law on state financial guarantee for provision of medical services in 2018, the revision of different services packages and the national rollout of the primary health care reform in 2020. All these reforms created a strong legal and political framework to implement new health financing arrangements and improve service delivery. A new payment mechanism was implemented for health care providers with a new purchasing agency to split the provider-purchased functions, while guaranteeing a package of health services with inclusion of the most prevalent NCDs. With the direct support of the health policy adviser, the Ministry of Health led several high-level policy dialogue meetings to ensure the required social cohesion to reform the national health sector. To support policy dialogues with credible data on health expenditure, WHO conducted a number of studies on the financial costs of health care in Ukraine. In addition, the country benefited technical assistance to establish an effective people-centred network of PHC providers. All these reforms were supported with provision of know-how, technical assistance and capacity building for translating the legislation into organizational setup, procedures, mechanisms and capacities to launch the health system transformation. With the extension of the war in 2022, health financing has been readjusted and PHC mobile teams deployed to ensure the continuity of efforts toward achieving Universal Health Coverage.					

## Actionable recommendations—Strategic and technical support to move toward UHC and health security

The WHO GPW13 supports a differentiated approach based on capacity and vulnerability to strengthen the integrated health system approach, which defines four different kinds of modalities for WHO support to Member States (49).

1. Policy dialogue to develop health systems in future for the more mature health system.
2. Strategic support to build high-performing systems in advanced health systems.
3. Technical assistance to build national institutions in more fragile health systems.
4. Service delivery to fill critical gaps in emergencies, when national and regional capacities are not able to maintain essential health services.

The UHC Partnership *de facto* contributed to developing this strategy, operating in countries for the second and third modalities, while always advocating for bottom-up, flexible, catalytic, and long-term support to Member States and implementing a new model of transparency and accountability (consistent and regular annual reporting, communication strategy, live-monitoring meetings, multi-donor, and internal three levels coordination mechanisms).

This strategy quickly brought interesting results in the formulation of public health laws, national strategies, road maps, and national compacts for UHC. Endorsed and acknowledged by the WHO senior management and partners, these results led to constitute a positive environment for the UHC Partnership, which grew from 30 countries to 115 between 2017 and 2020. The UHC Partnership played a key role in highlighting health system strengthening as a fundamental technical priority for WHO and other global health actors. It continues to remain an organizational priority (50), while its strategic approach, principles, and results are recognized by all WHO departments as well as financial and technical partners (51).

The first and most fundamental added value of the UHC Partnership is the long-term deployment of health policy advisers in WHO country offices. Health policy advisers are present in some countries for more than 10 years, and their positions are progressively integrated into the core workforce of the organization. They support the leadership of Ministries of Health in health policy-making processes for essential primary healthcare services and functions, according to WHO health-related guidelines, while convening national and international health stakeholders to build consensus around national health policies and orient human, financial, and technical resources to implement them.

Health policy advisers are senior generalist public health officers recruited to provide leadership and managerial support to country offices, as well as technical and policy advice to Ministries of Health, in the area of public health and health system strengthening, ensuring that the activities in these areas are carried out efficiently and effectively. They constitute the technical country reference for many technical areas and many partners with regard to health system strengthening. They are, for instance, involved in the development of PHC investment plans with the European Investment Bank, as primary providers of evidence and to coordinate technical discussions with National Authorities and partners.

In times of emergency, health policy advisers bring together all technical resources to ensure the continuity of essential health services, strengthen PHC for health security, including surveillance and treatment of diseases, and prevent routine local outbreaks from becoming larger disruptive emergencies. National health security plans can only be integrated into national health strategies to ensure that those specific functions to prepare, prevent, detect, and respond to disease outbreaks and other health emergencies are integrated based on basic health system functions and not separately.

Health policy advisers support the generation of evidence (34), for instance, the institutionalization of national accounts for health financing and workforce or the mapping of available resources and priority actions to increase preparedness capacities. They mobilize policymakers, civil society organizations, and international partners through evidence-based policy dialogues in order to reinforce strategic frameworks and increase resilience and coverage with essential health services, financial protection, and equity. Health policy advisers also encourage and support specific dialogues between Ministries, such as with the Ministry of finance to ensure the coherence and sustainability of the health budget according to national objectives, and to improve public financial management for health.

**Figure 3:**
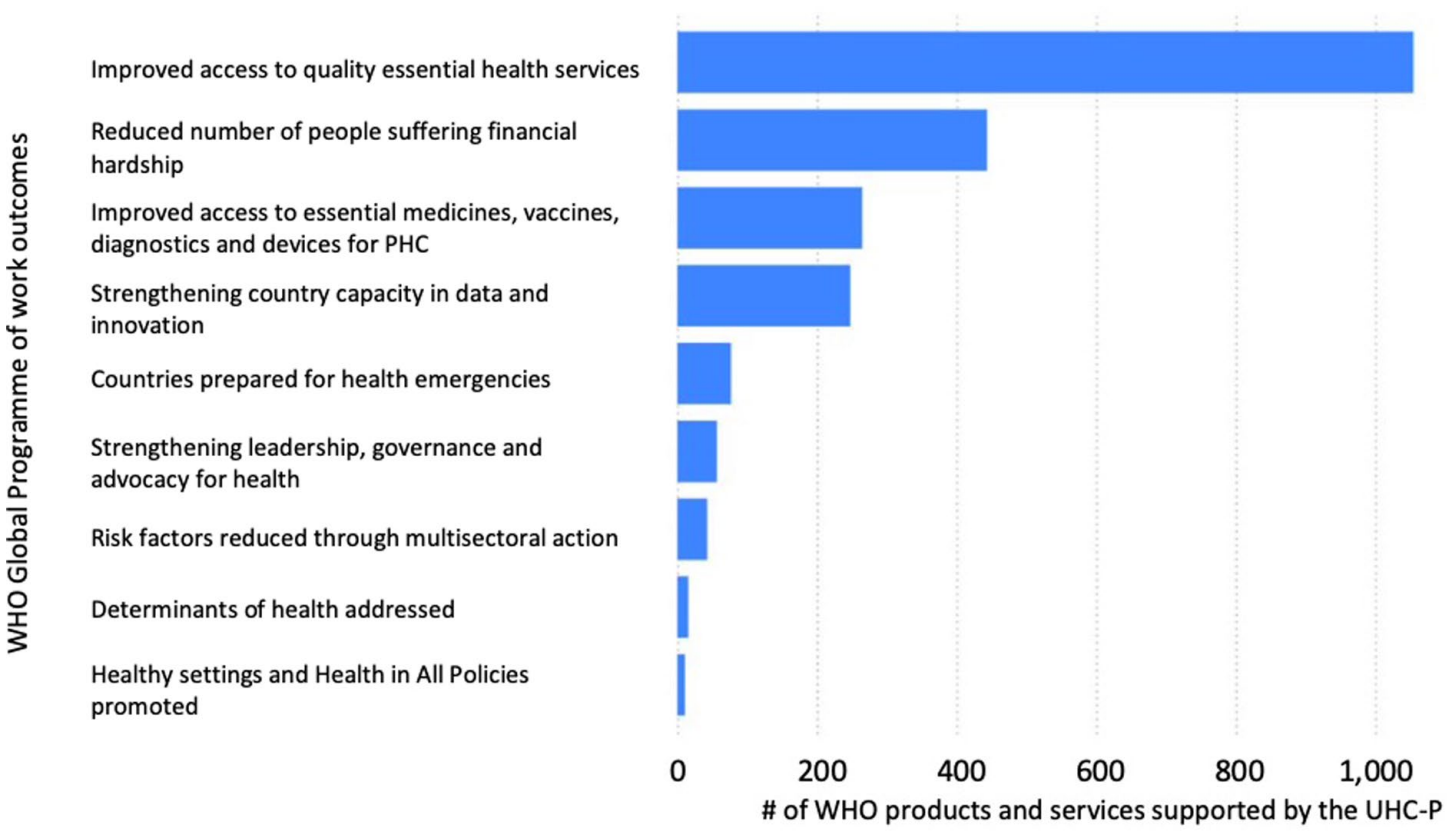
UHC Partnership support to the 13th WHO Global Programme of work, 2020-2021.

Policy dialogue between the Ministry of Health and other health stakeholders can lead to rationalizing the policy-making process with debates and decisions based on accurate representations of reality (52) and in the respect of international guidelines to strengthen Primary Health Care. This policy-making process can enable the alignment of health system objectives and resources to the needs of the population in order to make and sustain progress toward UHC and health security while enhancing social participation (27). Over the last decade, in many countries, road map, national compact, and legal frameworks for UHC and health security have been developed due to the support provided by the health policy advisers, according to the number of products and services supported by the UHC Partnership (Figure 3).

In 2021, a survey has been conducted among health policy advisers (*n* = 130) to understand their contribution to the COVID-19 response. Roughly 98% of respondents stated being in almost daily contact with their counterparts at the Ministry of Health. The survey indicated that, on average, respondents had to allocate 50% (range: 3–90%) of their full-time equivalent to support COVID-19-related response activities, albeit the significant amount of work planned under the frame of the UHC Partnership. In addition, due to the UHC Partnership’s flexibility, 90% of respondents were involved in and reinforced the in-country incident management support teams in response to COVID-19. Many of them (56%) even took up a specific position within the incident management support teams, either as an Incident Manager or as a lead or focal point for one of the components of the country’s strategic preparedness and response plan, particularly the pillar 9 on the maintenance of essential health services (53).

Through this network, the WHO has been able to extend its operational arm to bring coherent technical expertise to the Member States from the three levels and experiment with the transformation of the organization. Health policy advisers have enabled WHO country offices to strengthen technical support to Ministries of Health, other National Authorities, as well as Global Health partners by building technical capacities to lead health policy cycles and generate political commitment while creating synergies and harmonization between stakeholders and funding streams. Health policies can then be translated into processes, functions, and services to operationalize UHC, ensure Health Security, and serve population needs. Health policy advisers tend to reinforce all essential public health functions to ensure the minimum requirements to operationalize the right to health, one of the first responsibilities of Member States under the Universal Declaration of Human Rights and the constitution of the WHO.

Health policy advisers are described as crucial assets by WHO Regional and Country Offices in the integration of health systems to enhance their resilience through fostering coherence between essential public health functions and health outputs, always considering the social, economic, and political environment. Similarly, they are designated by the vertical program experts as key players to highlight the importance of integration of programs and provide related support, to move forward the UHC and SDG agenda in countries. The flexibility to adapt their terms of reference to each context and their continuous and long-term presence allow them to monitor policy processes, support technical analysis and participate in policy monitoring and evaluation processes, and use every opportunity to improve health governance. With their support, national authorities, WHO countries, and regional offices are defining actions to be implemented in order to welcome innovations and design theories of change fit for the context.

## Discussion

It is now increasingly clear for scholars that political economy is fundamental to understanding the appropriate ways for the implementation of UHC, health security, or essential public health functions as a political exercise (54), but also that “the political routes to UHC are diverse” (55). The WHO also acknowledged that health is primarily a political choice (56) and that a social contract for UHC and health security (27) is needed to ensure its implementation. Experiences from the UHC Partnership tend to confirm these hypotheses, demonstrating how this social contract can be renewed or built through evidence-informed policy dialogue mechanisms including all voices of the health system (57). In Timor-Leste, for instance, the institutionalization of the National Health Sector Coordination Committee leads to open a permanent health forum to oversee and discuss health policies and the implementation of projects and programs guided by one National Health Strategic Plan for all partners and stakeholders.

For a decade, health policy advisers funded by the UHC Partnership played the significant role of convener and broker to support key decision-makers in countries to develop UHC in their social, economic, and health policies for essential public health functions and align stakeholders and resources behind it. The work of the UHC Partnership around governance aims to integrate each essential public health function within its political environment. As demonstrated through multiple accountability mechanisms, supported policy dialogue in many countries has been leading to put UHC and health security on the political agenda and to develop integrated systemic and programmatic policies through the PHC approach (39, 40).

Due to the role of health policy advisers, the WHO is implementing activities that illustrate how the policy-making process for Primary Health Care can be supported in the country. These activities aimed to influence contextual factors (governance, financial and delivery arrangements, institutions, interests, ideas, and external factors) that are shaping health policies (58). The example of Ukraine, for instance, illustrates how the technical assistance contributed to a major reorganization of the health system and especially with regard to the financial and delivery arrangements through the establishment of new payment mechanisms with a National Health Purchasing Agency and a State Guaranteed Benefit Package for Primary Health Care.

John Kingdon’s concept of the window of opportunity (59) could be used to reflect and analyze the approach of the WHO. This classical policy-making model theorizes the setting of public policy agenda, as the intersection of three specific streams related to problem, policy, and politics. This intersection would open a window of opportunity for political decision-making and key reforms. The approach of the WHO to strengthen health systems could be described similarly.

While advocating for a PHC approach to reach UHC and health security, the WHO, through health policy advisers, makes positive propositions of concrete alternative policy and mobilizes policymakers to engage in reforms. Opening windows of opportunity for policy change based on renewed or innovative commitments, the WHO works on fundamental contextual factors for the health policy-making process to ensure that global or country-based strategic frameworks are in place to finally promote health, serve the vulnerable, and keep the world safe. In this perspective, the establishment of the Health Sector Stabilization and Recovery Plan in South Sudan aimed, for instance, to give a common framework to national authorities, humanitarian, and development actors in supporting the health system to move from an emergency situation to long-term development of the health sector.

The flexibility and the long-term presence of health policy advisers are critical to ensure that technical capacities are available when a window of opportunity for the policy-making process is opening, therefore, enhancing the presence and the operational capacities of the WHO. This was especially the case during the COVID-19 pandemic, where health policy advisers were immediately available to provide support to national authorities. Over the 10 years, the WHO has been able to create and sustain one of the largest and most effective platforms for international cooperation on Primary Health Care for UHC and health security. In 115 countries, the WHO has demonstrated what can be achieved through the reinforcement of strategic and technical leadership for health system strengthening and resilience attributable to a PHC-integrated approach, including more recently in the context of a pandemic and health emergencies.

In 2021, the WHO was the subject of the result-oriented monitoring (ROM) review by the European Commission. The role of health policy advisers has been especially distinguished to strengthen WHO support to Member States and deliver high-quality outputs in developing, implementing, and/or strengthening policies and actions of public institutions for health. The need for long-term partnership and financing support for the health reform process is also acknowledged, and the report finally recommends ensuring the sustainability of the intervention through the implementation, monitoring, and evaluation of health policies built during the first phases. The COVID-19 pandemic has nevertheless demonstrated that efforts to strengthen health systems are still mostly fragmented and do not ensure adequate commitment to or resourcing of essential public health functions to enable resilience, safeguard health, and insulate essential health service delivery.

However, as noticed in the 2019 UHC global monitoring report (60), all countries benefiting from dedicated technical assistance, through health policy advisers for health system strengthening from the WHO, have seen an increase in their UHC index during their involvement in the UHC Partnership prior to the COVID-19 pandemic. This progress is the result of the global movement for UHC and can be attributed to the National Authorities with the support of international and national health partners, including the contribution of WHO’s support on policy and strategic aspects for PHC and UHC.

This policy and practice review seeks to trace the first steps of longer research to understand the contribution of the UHC Partnership to the achievement of the Sustainable Development Goal target 3.8 for Universal Health Coverage. Available data, through the diverse accountability mechanisms of the UHC Partnership, have been adequate to demonstrate the contribution of the UHC Partnership to the institutionalization of health policy and strategies for PHC and UHC. The positioning of health policy advisers to provide direct in-country strategic and technical support to Members States, based on their needs, priorities, and strategies, is clearly a key actionable recommendation that needs to be duplicated and intensified to support the achievement of Universal Health Coverage.

However, these data are insufficient to establish a clear linkage between the activities supported by the UHC Partnership and the quantitative indicators 3.8.1 and 3.8.2. To establish stronger causality relations and introduce more reflexivity, a meta-narrative review (61) and deeper country case studies (35) could support a contribution analysis (62) during the next phase of the UHC Partnership. Moreover, the young and promising field of social epistemology demonstrates how political systems are shaping the distribution of population health (63). In an attempt to bridge political sociology and epidemiology (64), this discipline could provide relevant concepts and theories to understand the impact of the UHC Partnership on the social organization of power for health, and especially on health inequities, by supporting policy dialogue and including communities and minorities in policy-making processes.

## Conclusion

For more than 10 years, the UHC Partnership has been supporting the establishment of health policies and strategies to elaborate solid health systems foundations for primary care and essential public health functions. As some countries still suffer severe foundational gaps, additional and complementary technical expertise is required to continue the development of health policies and operationalize UHC frameworks and National Actions Plan for Health Security. In addition, aid coordination, domestic resources mobilization, and improved public financial management can orient adequate assets to initiate financial protection services, the supply chain of essential health products, and the development of basic infrastructure for health.

**Figure 4:**
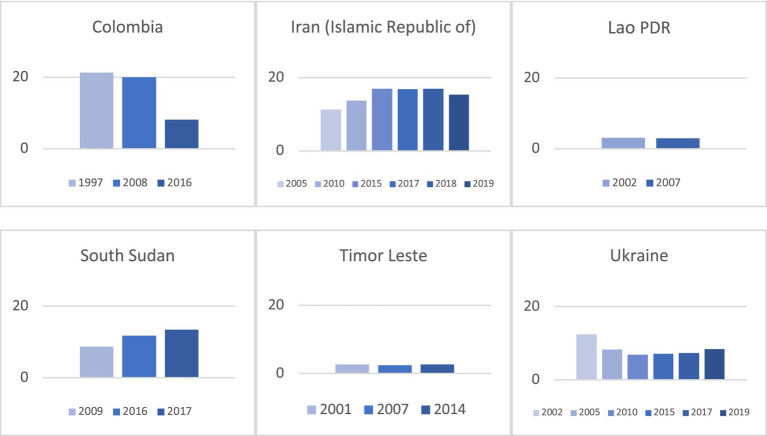
Evolution of the percentage of the total population with household expenditures on health >10% of total household expenditure or income (SDG 3.8.2).

**Figure 5:**
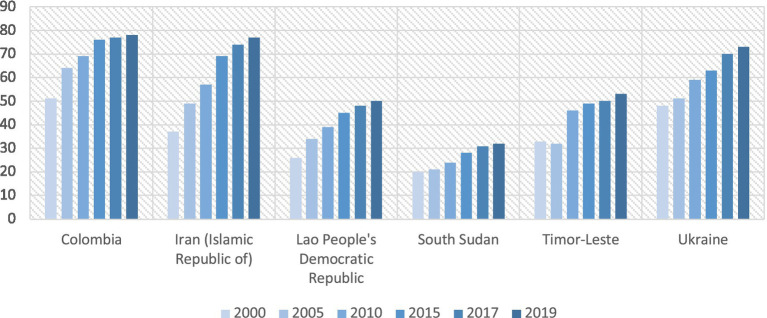
Evolution of UHC service coverage index in countries (SDG 3.8.1).

The 2021 UHC global monitoring report (65) revealed that, prior to the pandemic, improvements in service coverage were driven by massive investments to tackle communicable diseases. While much work remains to be done, especially with regard to financial protection (Figure 4), we need to recognize the progress achieved by many countries in improving their UHC service coverage index toward very ambitious targets (Figure 5). On the other hand, the percentage of the total population with households’ expenditures on health continues to be excessive and strong barriers remained, limiting access to healthcare for all, such as poor infrastructure without basic amenities, high level of out-of-pocket payments, shortages of health workers, or the unavailability of good quality pharmaceutical products.

Countries are in need to sustain the acceleration of their journey to UHC and health security. Such effort can benefit from the experiences and lessons learned from countries supported by health policy advisers and can be readily applied when governments implement their recovery plans. Furthermore, the COVID-19 experience has been a trigger for politicians and the public, in general, to further realize and understand the inexplicable linkages among health, socioeconomic development, and whole-of-society constraints.

Public health agencies, and particularly the WHO as a lead health organization, have an important role and responsibilities combined with development banks and multisectoral partners in reinforcing strategic and technical leadership for primary healthcare services and essential public health functions, especially in countries that still suffer from foundational gaps in terms of infrastructures, basic commodities, health financing, or health workforce, for instance. This includes institutionalizing mechanisms for the integration of efforts in health systems strengthening and health security as well as for multisectoral and multi-actor involvement with political commitment and resources for sustainability.

Following the 75th World Health Assembly, the WHO committed to increasing its budget for intensified PHC support to Member States and called for a radical reorientation of health systems toward PHC (66). This will only be possible if all health actors and organizations engage, align, and accelerate the movement to increase strategic and technical leadership, to strengthen health systems, and to make UHC and health security a reality for all.

## Author contributions

All authors listed have made a substantial, direct, and intellectual contribution to the work and approved it for publication.

## Conflict of interest

The authors are working in the Special Program on PHC of the World Health Organization, which hosts the Universal Health Coverage Partnership. The publishing costs are funded by the World Health Organization. The authors are staff members of the World Health Organization. The authors alone are responsible for the views expressed in this article, and they do not necessarily represent the decisions, policies, or views of the World Health Organization.

## Publisher’s note

All claims expressed in this article are solely those of the authors and do not necessarily represent those of their affiliated organizations, or those of the publisher, the editors and the reviewers. Any product that may be evaluated in this article, or claim that may be made by its manufacturer, is not guaranteed or endorsed by the publisher.
